# Infrared Properties and Terahertz Wave Modulation of Graphene/MnZn Ferrite/p-Si Heterojunctions

**DOI:** 10.1186/s11671-017-2250-2

**Published:** 2017-08-08

**Authors:** Dainan Zhang, Miaoqing Wei, Tianlong Wen, Yulong Liao, Lichuan Jin, Jie Li, Qiye Wen

**Affiliations:** State Key Laboratory of Electronic Films and Integrated Devices, University of Electronic Science and Technology of China, Chengdu, 610054 China

## Abstract

MnZn ferrite thin films were deposited on p-Si substrate and used as the dielectric layer in the graphene field effect transistor for infrared and terahertz device applications. The conditions for MnZn ferrite thin film deposition were optimized before device fabrication. The infrared properties and terahertz wave modulation were studied at different gate voltage. The resistive and magnetic MnZn ferrite thin films are highly transparent for THz wave, which make it possible to magnetically modulate the transmitted THz wave via the large magnetoresistance of graphene monolayer.

**Electronic supplementary material:**

The online version of this article (doi:10.1186/s11671-017-2250-2) contains supplementary material, which is available to authorized users.

## Background

Infrared (IR) and terahertz (THz) devices are highly important for many electronic systems such as radar [1], wireless communication [2], and security systems [3]. Thus it is critical to explore the materials [4–7] and structures [8–14] that can be used in the infrared and terahertz range. Recently it is found that the transmission of THz wave can be modulated with graphene field effect transistor (GFET) by tuning the intraband transitions of graphene monolayer [8]. In their original GFET THz modulator, B. Sensale-Rodeiguez and coworkers use 92 nm SiO_2_ as the gate dielectric material, which achieved modulation depth of 15% and modulation speed of 18 Kb/s of THz wave [8]. D. Zhang and coworkers investigated the optical THz modulation of graphene/SiO_2_ (150 nm)/p-Si GFET, which can be tuned by gate voltage [15].

Later, it was found that the THz wave modulation of GFET could be improved by replacing the gate dielectric with high-k and dense Al_2_O_3_ thin film, which is grown by atomic layer deposition [16]. Modulation depth of 22% and speed of 170 kHz was achieved in the graphene/Al_2_O_3_ (60 nm)/p-Si GFET by varying the gate voltage [16]. The improved modulation is attributed to the reduced Coulomb impurity scattering and cavity effect [16]. Further, by using Bi-doped YIG (k ~12.0) as dielectric materials in the graphene/Bi:YIG (50 nm)/p-Si heterostructure, modulation depth of 15% and speed of 200 kHz were achieved from 0.1 to 1.2 THz by applying gate voltage [17].

According to previous studies, dielectric layer can largely affect the performance of GFET that was used for THz and infrared wave devices. By carefully screening the dielectric materials, it is possible to tune the performance of GFET. In prior studies, nonmagnetic high-k dielectric layers were used for terahertz and infrared GFET devices, where electrical signal is extracted or applied. However, bifunctional magnetic and dielectric layers have not been studied for GFET for terahertz and infrared applications, which could be tuned by external magnetic field. Here, we introduce 150 nm sputtered MnZn ferrite thin films as the dielectric materials of GFET for THz and infrared applications. As a high-k [18] and magnetic materials, MnZn ferrite thin films could perform as an excellent dielectric layer and also introduce new functionalities in the GFET THz and infrared devices. Response of the graphene/MnZn ferrite/p-Si GFET to the infrared illumination was observed by comparing the I-V curves with and without infrared illumination at different gate bias. Meanwhile, electrical modulation of THz wave was achieved by the GFET as the gate voltage was varied. Subtle change of transmitted THz wave was also observed as the external magnetic field was varied.

## Methods

Mn_1-x_Zn_x_Fe_2_O_4_ thin films were prepared by RF magnetron sputtering. The target material was produced by co-precipitation of Fe(NO_4_)_3_, Mn(NO_4_)_3_, and Zn(NO_4_)_2_ solution, which is calcined at 950–1000 °C for 2 h, then pressed into a 60-mm disc, and finally sintered at 1250 °C for 3.5 h. The films were deposited on (100) p-Si substrates at 200–300 °C under base pressure of 4 × 10^−4^ Pa and oxygen concentration of 0–25% (P_O2_/(P_O2_ + P_Ar_)). The film (150 nm) was annealed in vacuum between 400 and 700 °C under pressure of 0.08 Pa–5.0 Pa for 1.5 h.

The crystal structures of Mn_1-x_Zn_x_Fe_2_O_4_ thin films were characterized using Cu Kα X-ray diffraction (XRD, D/max 2400 X Series X-ray diffractometer, Tokyo, Japan) at 40 kV and 100 mA. The microstructures of the Mn_1-x_Zn_x_Fe_2_O_4_ thin films were investigated using a scanning electron microscope (SEM: JOEL JSM6490LV). The surface arithmetic average roughness (Ra) and root mean squared roughness (RMS) have been measured by an atomic force microscope (AFM: Veeco Mutimode Nano4). The saturation induction was tested by an Iwatsu BH analyzer (SY8232). The magnetic properties of the films were measured by a vibrating sample magnetometer (VSM, MODEL: BHV-525).

**Scheme 1:**
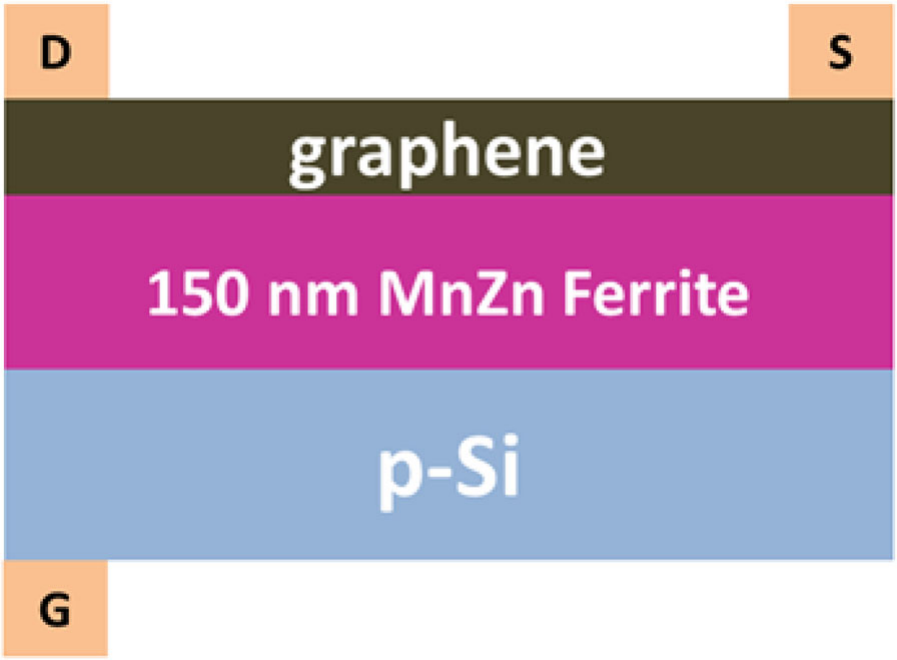
The GFET using 150 nm MnZn ferrite thin film as the gate dielectric material

After optimizing the growth conditions of Mn_1-x_Zn_x_Fe_2_O_4_ thin films on p-Si, graphene monolayers were then transferred from copper foil onto the Mn_1-x_Zn_x_Fe_2_O_4_ thin films to form graphene/MnZn ferrite/p-Si heterostructures. Graphene was fabricated by chemical vapor deposition (CVD) method in a tube furnace [19]. The transfer method of graphene monolayer was adapted from reference [20]. To fabricate the GFET, the electrode of gate, source, and drain was deposited by gold evaporation. The structure of the GFET using MnZn ferrite as gate dielectric material is shown in Scheme 1. The GFET was then characterized by a semiconductor parameter analyzer (Agilent 4155B) with a probe station (SUMMIT 1100B-M). For IR characterization, the I-V curves was measured under the IR illumination (*λ* = 915 nm, *P* = 1 W), which was compared with that in the dark environment. Terahertz wave transmission was measured by a THz time domain (TDS) system upon application of gate voltage and/or external magnetic field. The external magnetic field is generated by a home-made copper coil.

## Results and Discussion

**Fig. 1:**
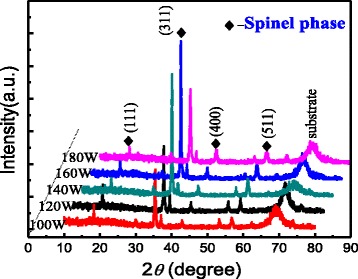
XRD patterns of samples on p-Si(100) substrate and sputtered under different RF magnetron sputtering powers 100, 120, 140, 160, and 180 W

Figure 1 shows the XRD patterns of the Mn_1-x_Zn_x_Fe_2_O_4_ ferrites thin films on p-Si (100) substrates sputtered under RF powers of 100, 120, 140, 160, and 180 W, respectively. Spinel structure of MnZn ferrite thin films was obtained under different sputtering powers. The (311) diffraction peak is the strongest, indicating the best crystallinity at deposition power of 160 W. Table 1 shows the surface arithmetic average roughness (Ra) and root mean squared roughness (RMS), and the length and width of maximum grains of the ferrite films on the p-Si (100) substrates. As shown in Table 1, the surface roughness (Ra and RMS) of the MnZn ferrite thin films increases with the RF power. However, very low RF power will affect the formation of MnZn ferrite thin films. The roughness of the MnZn ferrite thin films would affect the performance of the GFET IR and THz devices, which we discuss later.

**Table 1:** The roughness and grain size of MnZn ferrite thin film deposited at different RF power

RF sputtering power (W)	Ra (nm)	RMS (nm)	The length of maximum grains (nm)	The width of maximum grains (nm)
90	53.70	64.96	250.19	40.84
100	65.24	81.35	156.36	21.30
120	80.26	93.91	230.11	24.48
130	74.56	88.51	347.03	38.31
150	75.07	90.62	533.74	34.34

**Fig. 2:**
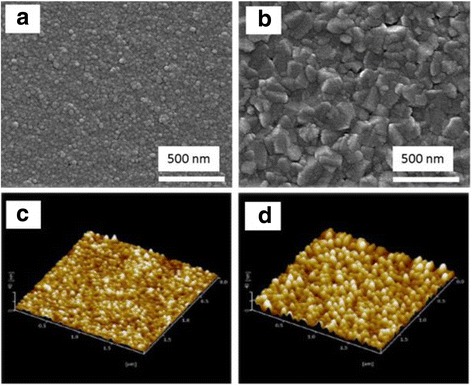
SEM images of (**a**) as-deposited and (**b**) annealed MnZn ferrite thin film, (**c**) and (**d**) show the corresponding AFM images

**Fig. 3:**
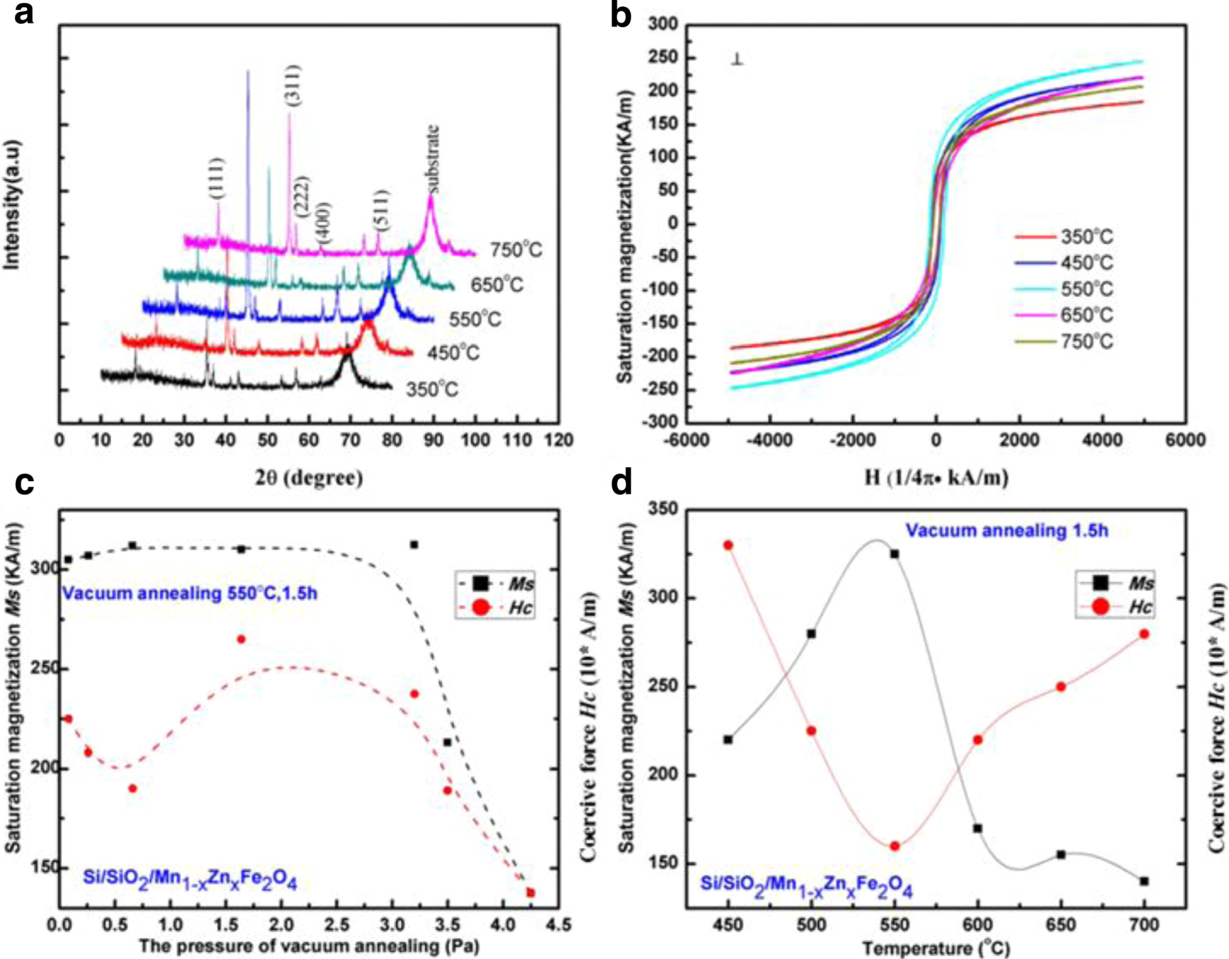
Characterization of sputtered MnZn thin films. (**a**) XRD patterns and (**b**) hysteresis loops of MnZn thin films annealed at 350, 450, 550, 650, and 750 °C. Saturation magnetization (*Ms*) of the MnZn thin films when annealed under the pressure from 0.0 Pa to 4.5 Pa in (**c**) and temperature from 450 to 700 °C in (**d**)

The SEM and AFM images of the MnZn ferrite thin films on p-Si substrates are shown in Fig. 2. The grains of MnZn ferrite thin films could be clearly observed. After annealed, the grain size increases as shown in Fig. 2b, d. Figure 3a shows the XRD patterns of the MnZn ferrite thin films annealed at different temperatures. The (311) peak of the MnZn ferrite thin film is the strongest when the film is annealed at 550 °C. The magnetic hysteresis loops of these thin films were also measured by VSM at room temperature and are shown in Fig. 3b, from which the saturation magnetization (*Ms*) and magnetic coercivity (*Hc*) are obtained. Figure 3c shows the *Ms* and *Hc* of the MnZn ferrite thin films annealed under the pressure of nitrogen gas up to 4 Pa. Below 3 Pa, the highest *Ms* and lowest *Hc* are obtained at 0.5 Pa. Above 3 Pa, the Ms decrease dramatically, which could be because of the reaction between nitrogen gas and the thin film. Figure 3d shows *Ms* and *Hc* of the ferrite thin film as a function of the annealing temperature at nitrogen pressure of 1.5 Pa. The *Ms* (*Hc*) value of the MnZn thin films reaches the maximum (minimum) value of 330 kA/m (1600 A/m = 20 Oe) at 550 °C. The maximum *Ms* and the minimum *Hc* corresponding the best crystallinity of the MnZn thin films, which in consistent with the XRD data in Fig. 3a. At higher temperature and gas pressure, the surface atoms of the thin film were nitrided into impurities, which deteriorate the magnetic properties of MnZn ferrite thin film. As a result, the MnZn thin films were prepared at annealing temperature of 550 °C and under vacuum pressure below 3 Pa.

**Fig. 4:**
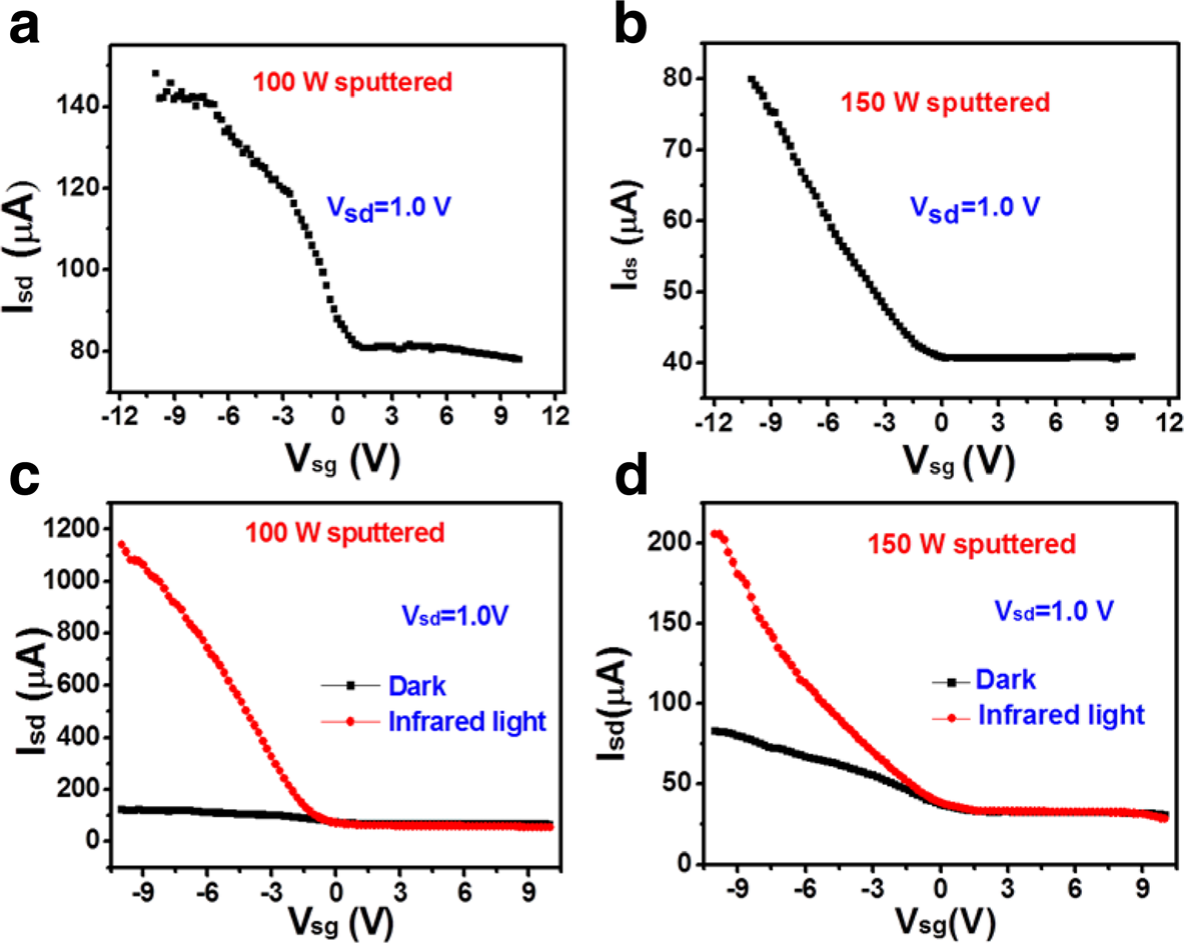
IR characterization. (**a**) and (**b**) I_sd_-V_sg_ curves of the GFET with MnZn ferrite thin film sputtered at 100 and 150 W, respectively. (**c**) and (**d**) compare the I_sd_-V_sg_ curves under IR illumination and no illumination. The voltage applied between source and drain is 1.0 V for all curves

Graphene grown on the same copper foil was then transferred onto MnZn ferrite thin films to make GFETs with structure shown in Scheme 1. Here, we fabricated GFET with MnZn ferrite thin films sputtered at 100 and 150 W and annealed in the optimal condition as discussed above. Figure 4a, b shows the electrical current measured between drain and source as a function of applied gate voltage for the two GFETs. During measurement, the applied voltage between source and drain is kept constant at 1 V. The current gradually increases as the gate voltage was negatively increased. The current changes very slowly when the gate voltage is positively biased. The asymmetric I-V characteristics of the two GFETs could be a result of the thermoionic emission and interband tunneling at the junctions between the gated and access regions [21]. The resistance of the graphene on the 100 W sputtered MnZn ferrite thin film is much smaller than that on the 150 W sputtered thin film at the same gate bias, as compared in Fig. 4a, b. The larger resistance in Fig. 4b could be a result of larger roughness of the 150 W sputtered MnZn ferrite thin films, as compared in Table 1. The roughness induced corrugation of the graphene monolayer could suppress the transport of charge carriers, leading to higher resistance [22].

Figure 4c, d shows the comparison of the I-V curves under dark environment and infrared illumination for GFETs using 100 and 150 W sputtered MnZn ferrite thin films, respectively. The infrared light is at wavelength of 915 nm and power of 1 W in a window of ~1 cm^2^. The applied voltage between source and drain is 1 V. The I-V curve of the GFET under infrared illumination is analogous to that measured in the dark environment, however, with significantly enhanced current. The enhancement is much stronger for the GFET using 100 W sputtered MnZn ferrite thin films as dielectric layer than that using 150 W sputtered MnZn ferrite thin film. The enhancement is ~7.5 times at gate voltage of 10 V for 100 W sputtered MnZn ferrite thin film, which is ~2.5 times for the 150 W sputtered MnZn ferrite thin film. Namely, the surface roughness of MnZn ferrite thin films could also affect the infrared optoelectronic properties.

**Fig. 5:**
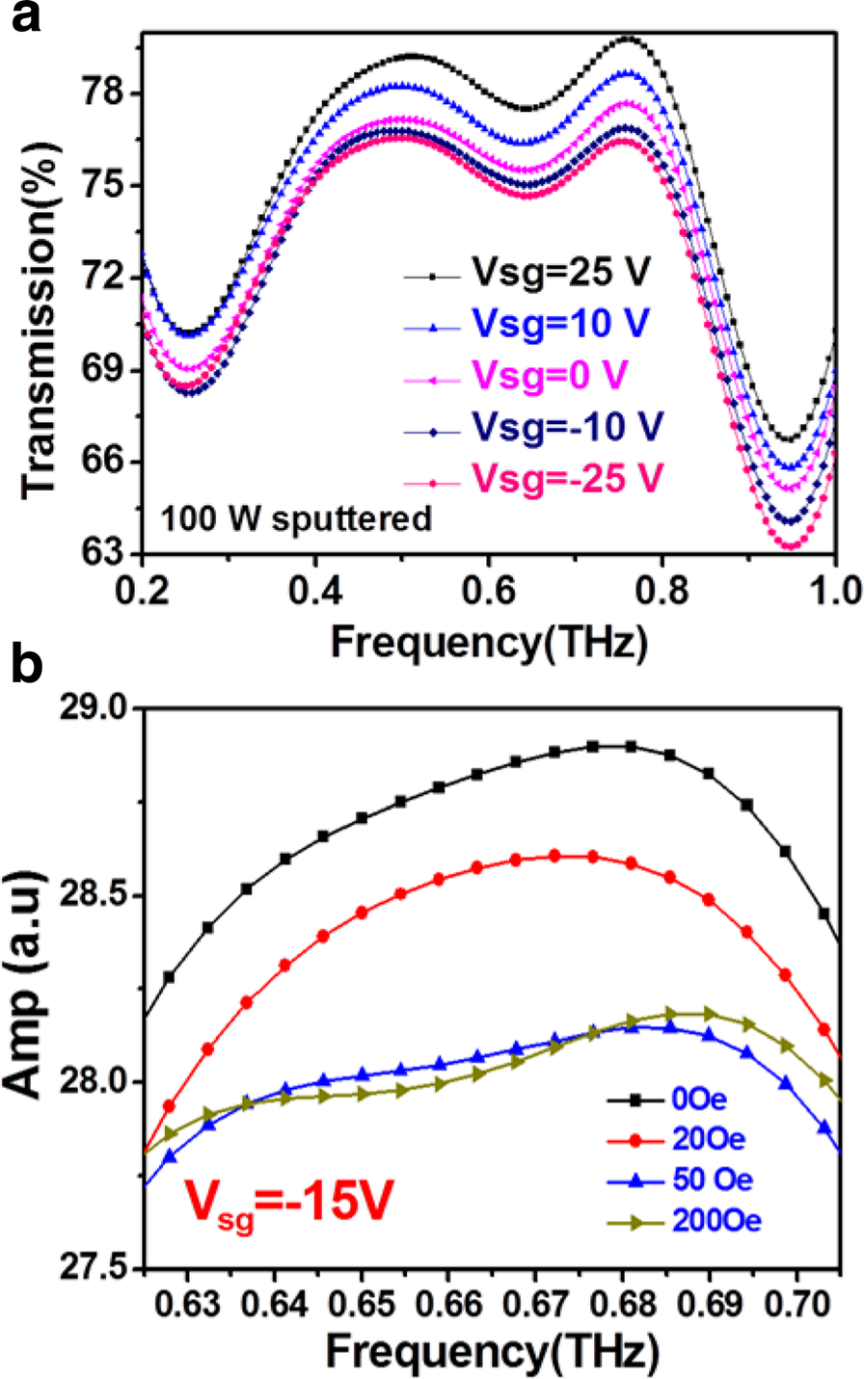
THz characterization. (**a**) The spectrum of THz transmittance from 0.2 to 1.0 THz at different gate voltages from −25 to 25 V, and (**b**) the frequency domain spectrum under different external magnetic field from 0.63 to 0.70 THz

The GFET with 100 W sputtered MnZn ferrite thin films was then used to examine the modulation properties of THz waves. Figure 5a shows the transmittance of THz waves through the GFET upon application of different gate bias. The transmittance was measured by a THz pulse using a THz-TDS system, and the transmittance in the frequency domain was obtained by Fourier’s transformation using air as the baseline. When the gate voltage is varied from 25 V to −25 V, the resistance between the source and drain is decreased, as shown in Fig. 4a.The reduction of resistance results in the reduced transmittance of THz wave, as shown in Fig. 5a. Namely, the transmission of THz wave could be modulated by applying different gate voltage of the GFET. The transmitted THz wave was also measured when an external magnetic field was applied, which is shown in Fig. 5b. As external magnetic field increases, the intensity of transmitted THz wave decreases, which saturate above 50 Oe. The change of transmitted intensity of THz wave under external magnetic field could be due to the extremely large magnetoresistance of graphene [23]. The underneath MnZn ferrite thin film provides strong fringe field upon magnetization by external magnetic field. The magnetoresistance of the graphene/MnZn ferrite/p-Si hetrojunction is shown in Additional file 1: Figure S1 in the supplemental information. However, the modulation of terahertz wave is subtle (5%), which could be because of the uneven surface of MnZn ferrite thin films and/or the small change of terahertz modulation with resistance. Graphene could feel much stronger and uniform fringe field on extremely smooth MnZn ferrite thin film, which could have larger magnetoresistance of graphene and give larger modulation depth by external magnetic field.

## Conclusions

Graphene/MnZn ferrite/p-Si heterostructure was fabricated for IR and THz device applications. The MnZn ferrite thin film was deposited on the p-Si by magnetron sputtering, which was annealed before used for GFET fabrication. The MnZn ferrite thin films provide an alternative dielectric material for the GFET IR and THz devices. As a magnetic and high-resistive thin film, it can strengthen the magnetoresistance of graphene and modulation of transmitted THz without introducing additional insertion loss. The surface roughness of the MnZn ferrite thin film can largely affect the performance of the IR and THz devices. Higher performance could be achieved by making MnZn ferrite thin film smoother. Such work is in progress.

## Additional file

Additional file 1: Figure S1. The magnetoresistance of graphene/MnZn ferrite/p-Si heterojunctions at Vsg = −15 V and room temperature. (DOCX 47 kb)
