# Supplementary material for: Infrared Properties and Terahertz Wave Modulation of Graphene/MnZn Ferrite/p-Si Heterojunctions
Source: Nanoscale Res Lett. 2017 Aug 8;12:482. doi: 10.1186/s11671-017-2250-2 (PMC5548700; doi:10.1186/s11671-017-2250-2)
Supplement: Additional file 1: Figure S1. — The magnetoresistance of graphene/MnZn ferrite/p-Si heterojunctions at Vsg = −15 V and room temperature. (DOCX 47 kb) [file 11671_2017_2250_MOESM1_ESM.docx]

Supplemental Information

Infrared properties and terahertz wave modulation of graphene/MnZn ferrite/p-Si heterojunctions

Dainan Zhang^1^, Miaoqing Wei^1^, Tianlong Wen^1*^, Yulong Liao^1^, Lichuan Jin^1^, Jie Li^1^, Qiye Wen^1^

1. State Key Laboratory of Electronic Films and Integrated Devices, University of Electronic Science and Technology of China, Chengdu, 610054, China

*E-mail: halong@uestc.edu.cn


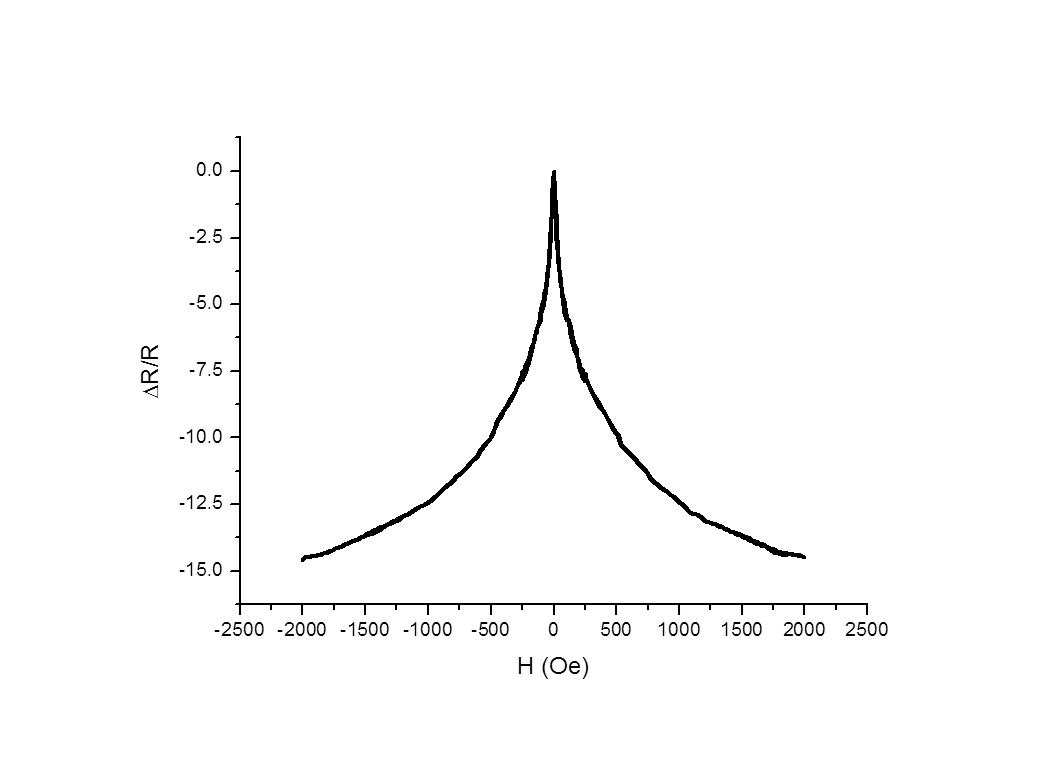


Figure 1S The magnetoresistance of graphene/MnZn ferrite/p-Si heterojunctions at Vsg=-15 V and room temperature.
